# Evaluating a preoperative protocol that includes magnetic resonance imaging for lymph node metastasis in the Cholangiocarcinoma Screening and Care Program (CASCAP) in Thailand

**DOI:** 10.1186/s12957-017-1246-9

**Published:** 2017-09-20

**Authors:** Metha Songthamwat, Nittaya Chamadol, Narong Khuntikeo, Jadsada Thinkhamrop, Supinda Koonmee, Nathaphop Chaichaya, Jeffrey Bethony, Bandit Thinkhamrop

**Affiliations:** 1 Faculty of Public Health, Khon Kaen University, Khon Kaen, Thailand; 2 Department of Radiology, Faculty of Medicine, Khon Kaen University, Khon Kaen, Thailand; 3 Cholangiocarcinoma Screening and Care Program (CASCAP), Khon Kaen University, Khon Kaen, Thailand; 4 Liver Fluke and Cholangiocarcinoma Research Center, Khon Kaen University, Khon Kaen, Thailand; 5 Cholangiocarcinoma Center of Excellence, Faculty of Medicine, Khon Kaen University, Khon Kaen, Thailand; 6 Department of Surgery, Faculty of Medicine, Khon Kaen University, Khon Kaen, Thailand; 7 Department of Obstetrics and Gynecology, Faculty of Medicine, Khon Kaen University, Khon Kaen, Thailand; 8 Department of Pathology, Faculty of Medicine, Khon Kaen University, Khon Kaen, Thailand; 9 Data Management and Statistical Analysis Center (DAMASAC), Faculty of Public Health, Khon Kaen University, Khon Kaen, Thailand; 10 Department of Microbiology, Immunology, and Tropical Medicine, School of Medicine and Health Sciences, George Washington University, Washington, USA

**Keywords:** Cholangiocarcinoma, Magnetic resonance imaging, Lymph node metastasis, Accuracy, Preoperative protocol

## Abstract

**Background:**

Treatment planning especially liver resection in cholangiocarcinoma (CCA) depends on the extension of tumor and lymph node metastasis which is included as a key criterion for operability*.* Magnetic resonance imaging (MRI) offers a rapid and powerful tool for the detection of lymph node metastasis (LNM) and in the current manuscript is assessed as a critical tool in the preoperative protocol for liver resection for treatment of CCA. However, the accuracy of MRI to detect LNM from CCA had yet to be comprehensively evaluated.

**Methods:**

The accuracy of MRI to detect LNM was assessed in a cohort of individuals with CCA from the Cholangiocarcinoma Screening and Care Program (CASCAP), a screening program designed to reduce CCA in Northeastern Thailand by community-based ultrasound (US) for CCA. CCA-positive individuals are referred to one of the nine tertiary centers in the study to undergo a preoperative protocol that included enhanced imaging by MRI. Additionally, these individuals also underwent lymph node biopsies for histological confirmation of LNM (the “gold standard”) to determine the accuracy of the MRI results.

**Results:**

MRI accurately detected the presence or absence of LNM in only 29 out of the 51 CCA cases (56.9%, 95% CI 42.2–70.7), resulting in a sensitivity of 57.1% (95% CI 34.0–78.2) and specificity of 56.7% (95% CI 37.4–74.5), with positive and negative predictive values of 48.0% (95% CI 27.8–68.7) and 65.4% (95% CI 44.3–82.8), respectively. The positive likelihood ratio was 1.32 (95% CI 0.76–2.29), and the negative likelihood ratio was 0.76 (95% CI 0.42–1.36).

**Conclusions:**

MRI showed limited sensitivity and a poor positive predictive value for the diagnosis of LNM for CCA, which is of particular concern in this resource-limited setting, where simpler detection methods could be utilized that are more cost-effective in this region of Thailand. Therefore, the inclusion of MRI, a costly imaging method, should be reconsidered as part of protocol for treatment planning of CCA, given the number of false positives, especially as it is critical in determining the operability for CCA subjects.

**Electronic supplementary material:**

The online version of this article (10.1186/s12957-017-1246-9) contains supplementary material, which is available to authorized users.

## Background

Cholangiocarcinoma (CCA) is a primary hepatic malignancy that arises along the intrahepatic and extrahepatic bile ducts. It is the second most common liver cancer globally [1] and the most common liver cancer in the resource-limited setting of the northeastern provinces of Thailand [2], where its high incidence is due to risk factors unique to this tropical disease, i.e., the consumption of raw or undercooked cyprinoid fish, which are the intermediate hosts for the food-borne trematode *Opisthorchis viverrini* (Ov), one of the few carcinogenic pathogens [3]. When detected early, CCA is amenable to liver resection, which substantially improves survival [4, 5], with the median survival time of liver resection patients at 23 months compared with less than 8 months in inoperable patients [5]. However, due to the asymptomatic or non-specific nature of the symptoms associated with CCA during its early stages, this cancer is often detected at an advanced stage, with a concomitantly poor prognosis and dismal survival rate [5]. The Cholangiocarcinoma Screening and Care Program (CASCAP) was instituted in Northeast Thailand with the objective of conducting community-based ultrasound (US) screening programs for the early diagnosis of CCA, making liver resection a viable post-diagnostic option for the treatment of CCA [6].

As the objective of CASCAP is to increase survival from CCA by aggressively screening for this cancer by mobile US units in Ov-endemic areas, a critical component of this endeavor was to establish a preoperative evaluation protocol for liver resection for CCA, which would include factors such as (1) tumor infiltration beyond the second order bile duct branches, (2) tumor invasion of major vessels such as main hepatic artery or portal vein, and (3) the extent of lymph node metastasis (LNM) [7–15]. Due to its location in the hepatoduodenal ligament, CCA quickly metastasizes outside the liver via the lymph node system or perineural invasion [16, 17], even during the early stages of this bile duct cancer. While several methods are available for detecting LNM, CASCAP attempted magnetic resonance imaging (MRI) to scan for LNM as part of a preoperative evaluation protocol for CCA due to its speed and “accuracy” in other cancer contexts [18]. However, the accuracy of MRI for depicting LNM for CCA has to be studied for each individual cancer, and it has been poorly studied of CCA [19–21]. The assessment of LNM by MRI for CCA is seldom performed due to limited access to tertiary care centers in this resource-poor setting. Herein, we investigated the diagnostic performance of MRI for the detection of LNM, as a part of the preoperative evaluation protocol for CCA, compared to the “gold standard” of pathological confirmation of individuals suspected of CCA by lymph node resection.

## Methods

### Study setting: community-based risk stratification and ultrasonography

This study was based on data from CASCAP, which is a combined community and hospital-based cohort study conducted in Ov-endemic areas in Northeastern Thailand (www.cascap.in.th) and nine tertiary care hospitals in this same region. The CASCAP study is comprised of two cohorts. The first cohort is the “screening cohort,” which includes individuals who were risk stratified using criteria consisting of (1) their residence in Northeastern Thailand, (2) age of 40 years or older (inclusive), (3) previous reported infection with the carcinogenic liver fluke Ov, (4) previously reported treatment for Ov infection with praziquantel, and (5) self-reported consumption of raw or undercooked freshwater fish, the second intermediate host of Ov*.* Individuals considered at risk for Ov-induced CCA underwent liver US as detailed in Khuntikeo et al. [6]. The second cohort is a conventional cancer registry, where patients were suspected of CCA during a routine US from one of the nine participating hospitals.

### Magnetic resonance cholangiopancreatography

Individuals included in the present study included those enrolled in CASCAP who were suspected of CCA by US or clinical symptoms and had a confirmatory magnetic resonance cholangiopancreatography (MRCP) or computerized tomography (CT) scanning at one of nine tertiary care centers. The MRI used a 1.5–3.0 T system. Two different MRCP techniques were applied: a single-shot rapid acquisition with relaxation enhancement (RARE) and a multislice half-Fourier acquisition single-shot turbo spin echo (HASTE). The slabs of the single-shot RARE sequence were obtained on various planes (e.g., coronal, axial, and oblique) to allow for the optimal visualization of the bile ducts. The number of thick-slab acquisitions ranged from seven to nine per patient. Next, multislice HASTE images were obtained in the coronal and oblique planes. Each examination was performed during a single breath-hold.

The imaging parameters for the single-shot RARE sequence were as follows: repetition time of ∞, effective echo time of 1200 ms, echo train length of 240, flip angle of 150°, slab thickness of 70 to 90 mm, field of view of 300 to 340 mm, matrix of 240 × 256, and an acquisition time of 2.32 s. The imaging parameters for the multislice HASTE sequence were as follows: repetition time of ∞, effective echo time of 95 ms, echo train length of 128; flip angle of 150°, section thickness of 4 mm without a gap and 13 to 15 slices (range of coverage 52–60 mm), field of view of 300 to 340 mm, matrix of 240 × 256, and an acquisition time of 18 to 20 s. Fat saturation was used to reduce a strong fat signal during image acquisition. The total acquisition time for all of the imaging steps in the MR imaging were less than 15 min.

### LNM determination

The determination of LNM by MRI was made by a consensus between two board-certified radiologists using the following morphological criteria: nodal size greater than 10 mm in short axis diameter, central necrosis, and inhomogeneous enhancement following intravenous contrast medium injection [22–24]. In equivocal cases, a gastrointestinal radiologist was also consulted. The determination for surgery was attained by a consensus between the surgeon and the patient, after the counseling process was completed.

### Gold standard pathological evaluation of LNM

In the case when a surgical treatment occurred, the operation types and plans were discussed with the doctors before the procedure. The time interval between MRI scan and operation was about 1 month. Lymph node dissections were performed in case of the presence of an enlarged or abnormal consistency of the lymph nodes. These dissected lymph nodes were sent for pathological examination. The diagnosis of pathological LNM was done by two board-certified pathologists. The procedures included each pathologist performing a gross and microscopic examination of the tissue specimen separately with the pathological diagnosis and lymph node metastasis made by consensus. In case of differences in the diagnosis between the two pathologists, immunohistochemistry strains were used to determine a consensus between both pathologists.

### Data analysis

The descriptive statistics of patients and the type of tumor were presented as mean and standard deviations, such as age. Categorical variables were presented as numbers and percentages. A comparison between LNM diagnosis using an MRI scan and pathological diagnosis included the following parameters: sensitivity, specificity, positive predictive values (PPV), negative predictive values (NPV), false positive rates, and false negative rates. They were calculated along with their 95% confidence intervals (95% CI). These diagnostic parameters were presented as either the overall CCA or CCA stratified into intrahepatic, perihilar, and distal type based on the American Joint Committee on Cancer (AJCC) criteria. All statistical analyses were conducted using Stata13 (Stata Corp, College Station, TX, USA). Attached data sets 1-3 were used for all analysis (Additional files 1, 2 and 3).

### Ethical approval

This study was conducted according to the principles of Good Clinical Practice (Chapter 2 of the International Conference of Harmonized Tripartite Guideline for Good Clinical Practice), the Declaration of Helsinki, and the national laws and regulations about clinical studies. CASCAP was approved by Khon Kaen University Ethics Committee (HE551404) for Human Research and received written informed consent from all patient participants.

## Results

**Fig. 1:**
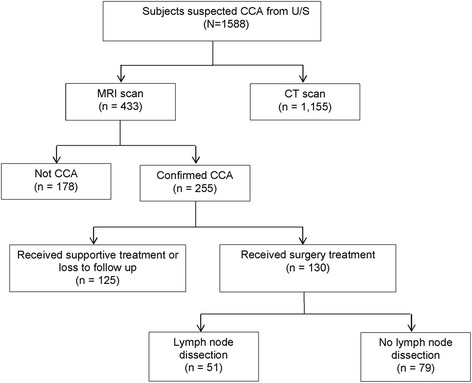
Flow of subjects in the Cholangiocarcinoma Screening and Care Program (CASCAP). Abbreviations: CCA cholangiocarcinoma, U/S ultrasonography, CT computerized tomography, MRI magnetic resonance imaging

A total of 433 individuals suspected of CCA by US were sent for MRI scanning, with a follow-up confirmation of 255 cases of CCA (58.9%). There were 133 (52.2%) individuals with intrahepatic CCA, 112 (43.9%) with perihilar CCA, and 10 (3.92%) with distal CCA. In the CCA group confirmed by MRI scans, 248 (97.3%) subjects also had radiographic lymph node findings, with 125 (50.4%) showing LNM. Of these 255 MRI-confirmed CCA subjects, 130 received surgical treatments with pathologically confirmed CCA. The 51 underwent lymph node dissection, with only 21 (41.2%) having confirmed LNM based on the conventional pathological diagnosis (Fig. 1).

Of the 51 individuals who underwent lymph node dissections, the mean age was found to be 61.5 years (SD 10.3), with 33 out of 51 (64.7%) being males. Of these individuals, 35.3% had intrahepatic, 60.8% perihilar, and 3.9% distal CCA (Table 1).

**Table 1:** Characteristics of patients and type of tumors presented as number and percentage unless specified otherwise

		Subjects	Percent
Mean age ± SD		61.5 ± 10.3 years	
Sex			
	Male	33	64.7
	Female	18	35.3
Type of tumor			
	Intrahepatic	18	35.3
	Perihilar	31	60.8
	Distal	2	3.9

*SD* standard deviation

The MRI scan accurately detected the presence or absence of LNM in 29 out of 51 CCA cases (56.9%, 95% CI 42.2–70.7), resulting in a sensitivity of 57.1% (95% CI 34.0–78.2) and a specificity of 56.7% (95% CI 37.4–74.5), with a PPV and a NPV of 48.0% (95% CI 27.8–68.7) and 65.4% (95% CI 44.3–82.8), respectively. The positive likelihood ratio was 1.32 (95% CI 0.76–2.29), and the negative likelihood ratio was 0.76 (95% CI 0.42–1.36) (Tables 2 and 3).

**Table 2:** Comparing the result of magnetic resonance imaging and pathological diagnosis (subjects)

		Lymph node pathology		Total
		Metastasis	Non-metastasis	
Magnetic resonance imaging scan	Nodal metastasis	12	13	25
	Non-nodal metastasis	9	17	26
	Total	21	30	51

**Table 3:** Diagnostic performance of magnetic resonance imaging in diagnosis of lymph node metastasis in different types of cholangiocarcinoma

	Diagnostic		Predictive value		Likelihood ratio	
CCA	Sensitivity (95% CI)	Specificity (95% CI)	Positive (95% CI)	Negative (95% CI)	Positive (95% CI)	Negative (95% CI)
Intrahepatic	50.0	88.9	80.0	66.7	4.50	0.56
	(23.0–72.2)	(51.8–99.7)	(28.4–99.5)	(34.9–90.1)	(0.63–32.9)	(0.27–1.17)
Perihilar	61.5	38.9	42.1	58.3	1.01	0.99
	(31.6–86.1)	(17.3–64.3)	(20.3–66.5)	(27.7–84.8)	(0.57–1.77)	(0.40–2.43)
Overall	57.1	56.7	48.0	65.4	1.32	0.76
	(34.0–78.2)	(37.4–74.5)	(27.8–68.7)	(44.3–82.8)	(0.76–2.29)	(0.42–1.36)

*CCA* cholangiocarcinoma, *CI* confidence interval

For intrahepatic CCA, the MRI scan had a sensitivity of 50.0% (95% CI 15.7–84.3) and a specificity of 88.9% (95% CI 51.8–99.7), with a PPV and a NPV of 80.0% (95% CI 28.4–99.5) and 66.7% (95% CI 34.9–90.1), respectively. The positive likelihood ratio was 4.50 (95% CI 0.63–32.4), and the negative likelihood ratio was 0.56 (95% CI 0.27–1.17) (Table 3). In the case of perihilar CCA, the MRI scan had a sensitivity of 61.5% (95% CI 31.6–86.1) and a specificity of 38.9% (95% CI 17.3–64.3), with a PPV and NPV of 42.1% (95% CI 20.3–66.5) and 58.3% (95% CI 27.7–84.8), respectively. The positive likelihood ratio was 1.01 (95% CI 0.57–1.77), and the negative likelihood ratio was 0.99 (95% CI 0.40–2.43) (Table 3).

## Discussion

The age-adjusted incidence rates for CCA in Northeastern Thailand are 89.2 and 35.5 per 100,000 for males and females, respectively, which are among the highest in the world. The high incidence of this relatively rare cancer in Northeast Thailand is due to the dietary habits of the inhabitants of the region, who consume raw or undercooked cyprinoid fish, the intermediate host of the food-borne pathogen *Opisthorchis viverrini*. CCA tends to present late, often going undetected until an advanced stage, when overall survival is less than 12 months [4, 25–29]. Currently, the most successful treatment for CCA is radical liver resection at an early stage of the disease and in the absence of LNM. With its location in the hepatoduodenal ligament, CCA tumors tend to metastasize outside the liver either via the lymph node system or via perineural invasion [16, 17]. Health disparities in this region of Thailand exacerbate this poor prognosis, as there are limited early cancer screenings and minimal post-diagnostic treatment. CASCAP was instituted in Northeastern Thailand to resolve these health disparities associated with CCA by conducting community-based US screening to detect early CCA.

The development of a preoperative protocol for CCA is central to the strategy of CASCAP. Currently, the preoperative protocol for CCA includes an (1) extension of the cancer to the hepatic artery or portal vein and (2) extension of lymph node metastasis (LNM) [7–15]. CCA cases that underwent liver resection in the presence of pathologically confirmed LNM were reported to have a zero 5-year survival rate [30–33], whereas CCA cases that underwent liver resection in the absence of LNM were reported to have a 35–72% 5-year survival rate [33–36]. However, the specific outcome of liver MRI on the surgical management of CCA has yet to be systematically evaluated. While several studies have estimated the diagnostic accuracy of MRI as part of the preoperative evaluation of bile duct involvement and vascular involvement [19, 20, 37], our study is the first to have determined the accuracy of MRI in the preoperative evaluation of LNM in CCA.

In the current study, MRIs were observed to have limited utility for preoperative evaluation for CCA compared to the “gold standard” of pathological confirmation of LNM. An especially concerning finding was the high false positive rate of MRIs for CCA LNM, which would exclude individuals otherwise eligible for surgery. We quantified the sensitivity of MRI scans in determining LNM for CCA at 57.1%, which was far lower than other preoperative resectability criteria such as the bile duct and vascular involvement [19, 20, 37]. A factor that may limit the accuracy of MRI scans in the assessment of CCA LNM is the reliance on nodal size in the axial short axis because of the inability to detect microscopic metastasis in normal-size nodes and to distinguish benign enlargement from malignant lymph nodes [12]. Our findings are similar to a recent study that found the survival rate of CCA with preoperative lymph node enlargement improved after hepatectomy [38]. We also agree with a study that recommended routine lymphadenectomy in all cases of hepatectomy [36]. An important limitation of this study is that MRI scanning was carried out at nine different tertiary care centers, using different machines, techniques, and radiologists. This indicates the possibility of variation in the data collection. Second, the lymph node pathology, which is the gold standard of this study, was determined in only some surgical subjects: not all MRI scans confirmed CCA diagnosis. The site and number of lymph nodes from MRI and pathological diagnosis also might be different. This might have an effect on our results. Finally, the time between an MRI scan and surgical operation might have affected the progression of the disease, as it may affect the period during which LNM may occur.

Liver resection has long been shown to increase survival from CCA, though it is highly dependent on LNM. Herein, we utilized the unique early screening program and a large sample size (CASCAP) in Northeastern Thailand to determine the quality of data for MRI scanning to determine LNM in the preoperative evaluation for CCA. We found that MRI scanning for LNM had limited sensitivity, with a low positive predictive value, and a high number of false positives, which would define eligible patients as inoperable. The evaluation of the nodal metastasis based on this radiological imaging modality has significant clinical implications for the surgical treatment of CCA. As such, we strongly recommend a “reconsideration” of the inclusion of MRI results for LNM in the preoperative evaluation for CCA, especially when it is used as the single or principle criterion for whether or not an individual will have curative liver resection for CCA.

## Conclusions

MRI has limited sensitivity and positive predictive value for the diagnosis of LNM in CCA. Therefore, surgeons should be aware of the number of false positives in determining the inoperability of CCA subjects.

## Additional files

Additional file 1: Dataset 1. (CSV 16 kb)

Additional file 2: Dataset 2. (CSV 22 kb)

Additional file 3: Dataset 3. (CSV 52 kb)

## Abbreviations

AJCC: American Joint Committee on Cancer
CASCAP: Cholangiocarcinoma Screening and Care Program
CCA: Cholangiocarcinoma
CI: Confidence interval
CT: Computerized tomography
HASTE: Half-Fourier acquisition single-shot turbo spin echo
LNM: Lymph node metastasis
MRCP: Magnetic resonance cholangiopancreatography
MRI: Magnetic resonance imaging
NPV: Negative predictive values
Ov: *Opisthorchis viverrini*
PPV: Positive predictive values
RARE: Rapid acquisition with relaxation enhancement
US: Ultrasound
